# Mirror, mirror, on the wall: During pandemics, how can self-perception research in people with eating disorders happen at all?

**DOI:** 10.1186/s40337-022-00608-8

**Published:** 2022-06-30

**Authors:** Zhen An, Isabel Krug, Jade Portingale, David Butler

**Affiliations:** 1 Melbourne School of Psychological Sciences, The University of Melbourne, Redmond Barry Building, Level 7, Room 707, Melbourne, VIC 3010 Australia; 2 The Cairnmillar Institute, Hawthorn East, VIC Australia

**Keywords:** Self-perception, Embodiment illusion, Enfacement illusion, Body image concerns, Eating disorders, Anorexia nervosa

## Abstract

**Background:**

‘Classic’ embodiment illusions (e.g., the feeling of owning another person’s hand) involves a three-way interaction between visual, proprioceptive, and tactile stimuli. These illusions have been studied in eating disorder (ED) populations given the potential implications for better understanding and improving body image concerns. Recently, several studies have employed ‘tactile-reduced’ methods mainly relying on the integration of visual and proprioceptive information to induce embodiment illusions in non-ED populations. To date, there has been no substantial review of these tactile-reduced approaches to consider their potential research and clinical utility in ED populations.

**Method:**

This review sought to examine and integrate studies across three areas. Firstly, those that employed tactile-reduced embodiment techniques in non-ED populations. Secondly, those that used classic embodiment techniques to compare whether ED and non-ED populations differ in their susceptibility to embodiment. Thirdly, studies that investigated whether experiencing classic or tactile-reduced embodiment techniques can improve image-related concerns.

**Results:**

For the first aim five studies were identified, all of which found that tactile-reduced methods consistently induced embodiment illusions in non-ED individuals. For the second aim, seven studies comparing ED and non-ED samples were found. ED patients were more susceptible to embodiment than non-ED samples in four studies, less susceptible in one study, and equally susceptible in two studies. Finally, for aim three, amongst the seven studies that used classic or tactile-reduced embodiment methods in ED populations, six reported improvements in self-perception (i.e., reduced body size overestimation, decreased body dissatisfaction and fear of gaining weight).

**Conclusions:**

Along with the classic approach, tactile-reduced embodiment approaches have implications for ED research and clinical practice, particularly for situations when face-to-face contact with people are restricted. Suggestions are provided for future researchers who wish to ensure best practice for planning embodiment research involving classic and tactile-reduced approaches.

## Plain English summary

Embodiment illusions involve the temporary experience of owning another person’s body or body parts. These illusions have been studied in people with eating disorders (EDs) as a means for (i) better understanding their self-perception issues (e.g., they tend to see their own appearance as being too big), and (ii) improving these self-perception issues (i.e., does experiencing embodiment reduce distortions involving body size?). ‘Classic’ embodiment illusions typically require participants to simultaneously experience visual, proprioceptive, and tactile sensory stimulation. However, COVID -19 pandemic restrictions have limited research in face-to-face settings which poses a challenge for the classic approach, mainly in relation to tactile input which would usually be provided by a researcher. A ‘tactile-reduced’ approach for eliciting embodiment illusions would, therefore, enable this field of research to continue, which in turn may benefit people with EDs. While several studies in non-clinical populations have demonstrated the effectiveness of a tactile-reduced approach, understanding surrounding this approach in ED populations is lacking. In this review, we offer a prospective set of suggestions regarding how tactile-reduced embodiment may be conducted with ED populations, both in terms of better understanding their (problematic) self-perception, and how such concerns may be improved.

## Introduction

‘Embodiment’ refers to having an illusory sense of ownership over another body (part) external to one’s own (e.g., another person’s hand or a full body). Such illusions have typically resulted from participants simultaneously experiencing tactile stroking processes combined with visual and proprioceptive input (see below). Embodiment illusions have been studied in many clinical populations including eating disorder (ED) patients in both real and virtual settings as they might contribute to a better understanding of—and ultimately treatments for—clinical conditions involving body image concerns [1, 2].

The coronavirus disease 2019 **(**COVID-19) pandemic has highlighted potential difficulties within ED research: specifically, restrictions involving face-to-face interactions with research participants [3]. In a recent survey that investigated the impact of the current pandemic on 187 ED researchers, respondents expressed high concerns about data collection and recruiting participants, with 20–40% of their current projects being stopped [3]. Consequently, within the embodiment research domain, it is crucial to consider whether illusions without tactile stimulation (which remove the need for face-to-face interaction) can be used to further investigate ED patients. A ‘tactile-reduced’ approach that can be used online without any face to face interaction is important because research has shown that ED patients are especially vulnerable to the negative impact of the pandemic due to restricted specialized ED treatment access, social isolation due to continuous lockdowns, and the ED-related/anxiety-provoking media drawing greater attention to food, thin ideals and weight increases during the pandemic [4–6]. Accordingly, increased ED thoughts and decreased motivation to recover due to the pandemic have more recently been reported in ED populations [7]. This narrative review, therefore, seeks to focus on embodiment studies that employed tactile-reduced methods (i.e., mainly visual and proprioceptive stimulation) and how their findings may be applied to ED populations, particularly in light of what we already know about how classic embodiment illusions may reduce body image concerns in ED populations. Implications of these reviewed findings for further research and clinical practice involving ED patients—particularly in the contexts where face-to-face contact is limited (i.e., COVID-19)—will also be discussed.

### ‘Classic’ embodiment illusions

**Fig. 1:**
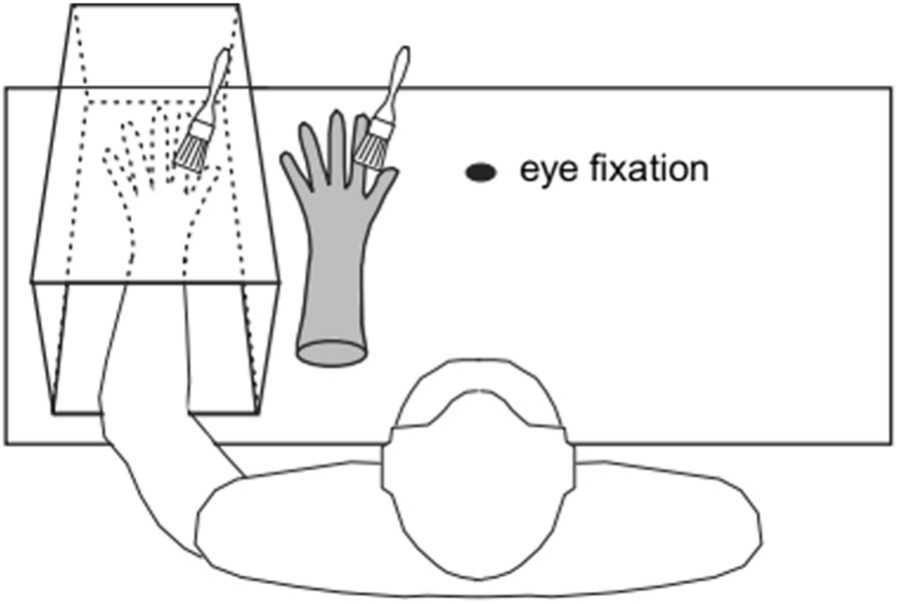
Example of the Procedure Used to Induce the Rubber Hand Illusion (used with permission from [10]. *Note.* The participant sees a rubber hand being stroked synchronously or asynchronously with their own occluded hand

Embodiment illusions are surprisingly easy to manipulate. The best-known example involves the ‘classic paradigm’ known as the rubber hand illusion (RHI; [8], see Fig. 1). In this illusion, participants are typically seated with one of their arms resting on a table with an opaque screen placed between their real hand and a rubber hand positioned on the table in front of them. After their own hidden hand and the rubber hand are synchronously stroked with brushes by an experimenter, participants quickly experience a sense of ownership and agency over the rubber hand.

**Fig. 2:**
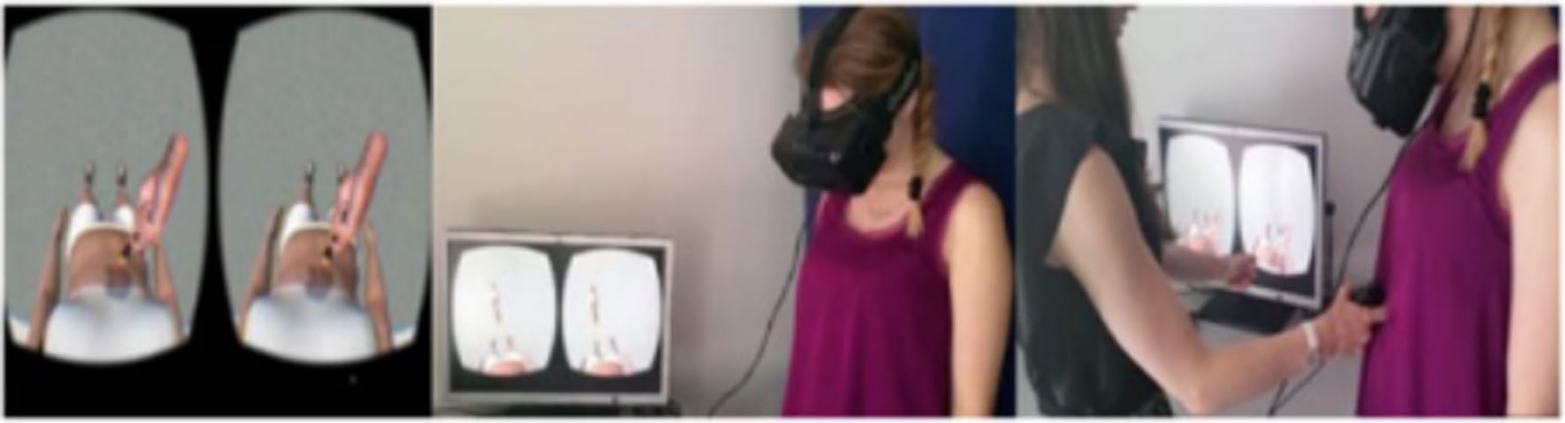
Example of the Procedure Used to Induce the FBI in a VR setting (used with permission from [12]. *Note.* The participant sees an avatar’s abdomen being stroked synchronously or asynchronously with their own abdomen from a first-person perspective via a head-mounted display in a VR setting

**Fig. 3:**
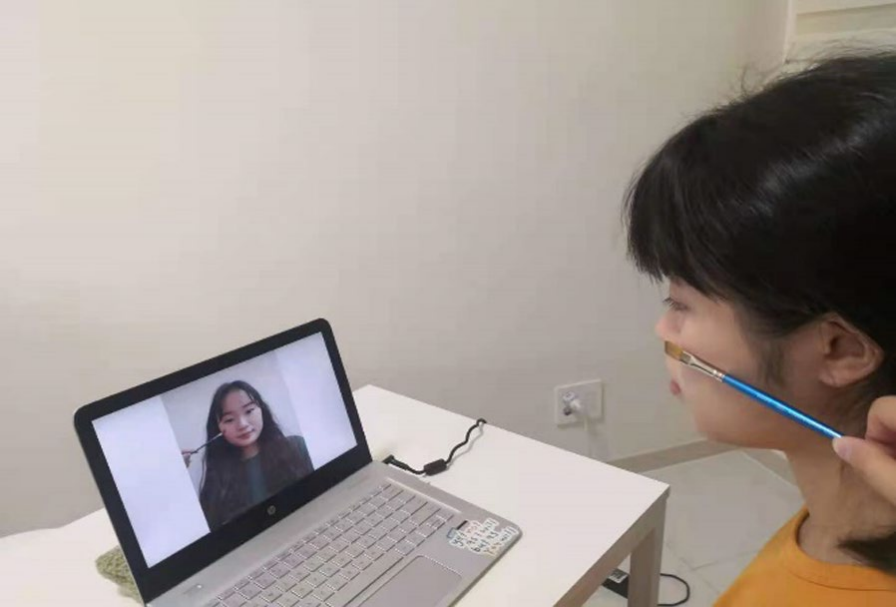
Example of the procedure used to induce the enfacement illusion. *Note.* The participant sees another person’s face in the screen being stroked by a paintbrush synchronously or asynchronously with their own face

Several variations of the original RHI procedure have been developed over the past 2 decades (for review see [9]). Among them, the full body and enfacement illusions target one’s full body and face, respectively. Full-body illusions (FBI) are often generated within a virtual reality (VR) environment (Fig. 2), whereas the enfacement illusion simply employs the process of watching another person’s face on a computer screen (Fig. 3). As with the RHI, full-body and enfacement illusions have typically been elicited by observing another model’s (e.g., an avatar’s or actor’s) body parts being stroked synchronously with one’s own corresponding body parts, with the latter not being visible to the participant (see Fig. 2). Common measures of embodiment used by researchers—both subjective and objective—are summarised in Table 1. Very briefly, subjective measures typically involve participants completing a Likert questionnaire, with responses indicating the extent to which they felt that the body part was their own, whilst objective measures tend to employ different variations of a perceptual task (e.g., estimation of body [parts] size pre and post embodiment).

**Table 1:** Common subjective and objective measures used for various embodiment illusions

Measures	Details
*Rubber hand illusion*	
Embodiment questionnaires [subjective] [1, 16, 26, 27, 31, 40]	Consist of between 2 and 10 items scored with Likert scales (7 or 10-points). These questions assess embodiment experiences including the perceived location, agency (control), and ownership over the hand Higher scores indicate a stronger experience of the embodiment illusion
Hand size estimation task [objective] [27]	For the rubber hand and their own hand, participants estimate (i) the width of the wrist, (ii) the width and the length of the hand. This is done before and after the RHI induction. For each size estimation, a calliper is used to let participants indicate when the hand fits exactly in between the two pointers of the calliper. This is done while the experimenter is moving the two pointers (1) away from each other and (2) towards each other along the back of the RHI set-up The estimation made after the RHI is subtracted from the one made before it; positive values indicate an increased size estimation after the RHI induction (i.e., greater embodiment)
Proprioceptive drift [objective] [1, 26, 27]	Participants indicate the location of their finger/hand before and after the RHI induction. For example, participants say ‘stop’ as soon as they think a vertical metal bar installed at the back of the RHI set-up matches the location of the middle part of their own finger [27]. During the task, their hands and the rubber hand are occluded from view Proprioceptive drift is calculated as the difference between the estimated location of the participants own finger/hand before and after the RHI. A positive value, or a larger bias in proprioceptive judgment towards the rubber hand is interpreted as the participant experiencing a larger visual dominance of the rubber hand over proprioception of their own unseen hand (i.e., greater embodiment)
Reaching task [objective] [31]	Participants view the upper half of a computer screen upon which a white target line appears in one of 15 locations after each RHI induction. Each line is equally spaced between the visible rubber hand and the participant’s own occluded hand, both of which are placed beneath the screen. The participants are instructed to use their own unseen hand to make an immediate response towards the target location that appears on the screen by touching the screen collinear with the target line below the viewing field ‘Reach endpoint errors’ are scored by calculating the difference between their movement endpoints and actual target locations (i.e., greater embodiment)
*Full body illusion*	
Body size estimation [objective] [28, 38, 39]	Participants are instructed to place two adhesive markers on the wall to estimate the width of their body (parts) before and after the FBI induction. Each marker represents the left and right side of the body (parts). Participants also estimate their circumference by using a piece of string/cotton rope Decrease in body (part) size overestimation indicates a stronger experience of the FBI
Embodiment questionnaires [subjective] [14, 28, 38]	Consist of between 6 and 20 items scored using a Likert scale (between 5 and 10). These questions assess embodiment experiences including the perceived location, agency (control), and ownership over the targeted body(part/s) Higher scores indicate a stronger experience of an embodiment illusion
Visual analogue scales [2, 30]	Participants estimate the intensity of the illusion from 0 to 100 on a visual analogue scale. Items range from 1 to 9 (although with the latter these items formed 3 subscales—ownership, agency, and referred touch—which were each scored separately, rather than as a total) Higher scores indicate a stronger experience of the FBI
*Enfacement illusion*	
Enfacement questionnaires [subjective] [21–23]	Consist of between 9 and 13 items scored with either Likert scales (between 7 and 10). The questions assess enfacement experiences including perceived agency (control) and ownership over the other’s (real or virtual) face Higher scores indicate a stronger experience of the enfacement illusion
Including Other in the Self scale [subjective] [23]	A single-item scale where self and other are represented by seven Venn-diagram-like pairs of circles. Participants choose the overlap that they think best represents the level to which the avatar’s face looks like their own Higher Venn values show a higher perceived self-other integration (i.e., higher enfacement)
Self-other discrimination morphing task [objective] [21]	Participants are shown morphing sequences (e.g., videos) which typically begin with the enfacement model’s face, and then gradually changes to the participant’s own face. The task involves participants to indicate when they feel that the face looks more like their own face rather than the model’s face. This task is usually done at baseline (before any enfacement procedure) and after experiencing synchronous or asynchronous enfacement Comparisons of participant’s choices between each condition with baseline indicates how much enfacement has occurred (i.e., they choose an image for self that contains higher levels of the model’s face compared to baseline), with larger changes indicating more enfacement
Self-face recognition morphing task [objective] [22]	Participants view a series of 9 static images that gradually transition from the enfacement model’s face to the participant’s face; each image has a different level of morphing between the participant’s and the model’s face (e.g., 80% participant vs 20% avatar). Participants are asked to indicate whether the image is the avatar’s or their own face by a key-press Comparisons of participant’s choices between each condition with baseline indicates how much enfacement has occurred (i.e., they choose an image for self that contains higher levels of the model’s face compared to baseline), with larger changes indicating more enfacement

### Explaining embodiment illusions: predictive coding theory

Embodiment illusions reveal important facets of self-perception: namely, that self-perception is not a static phenomenon, but rather involves a process by which we constantly update our mental representations about our appearance based upon multisensory inputs (e.g., visual, tactile, and proprioceptive inputs). The dominant theoretical explanation for embodiment illusions is known as predictive coding theory (for reviews see [10, 11]). Briefly, this theory proposes that (self) perception is a result of the brain comparing incoming sensory data with its own internal ‘predictive’ models based on past experiences about what is (or should be) happening. If sensory input is inconsistent with internal models, ‘prediction errors’ occur by which the brain attempts to re-align the incoming sensory input with the internal model, which in turn may result in illusory experiences. Therefore, the RHI can be interpreted as the result of minimising an apparent predictive error which occurs by updating one’s representations of their unseen real hand’s location and appearance to be more consistent with those of the rubber hand. Stated differently, the participant may infer “I feel my hand being stroked whilst seeing a rubber hand being similarly stroked at a place near where my real hand is located, therefore the rubber hand must actually be my hand”. This explanation can be applied to other types of embodiment illusions that target different body parts such as the full body and the face which are explored more below.

### Studying embodiment illusions through a tactile-reduced approach

Embodiment illusions have been repeatedly demonstrated via the ‘classic’ approach involving combined visual, proprioceptive, and tactile inputs (e.g., [12, 13]). Recently, however, it has been suggested that a simplified method merely involving combined visual and proprioceptive stimulation (hereafter referred to as ‘tactile-reduced’) could be sufficient to elicit embodiment illusions (see Tables 2 and 3 for a summary). For instance, having participants’ real bodies occluded from view by a mannequin’s headless body, Carey and colleagues [14] reported that merely observing the mannequin’s body from this first-person visual perspective (whilst wearing a head-mounted display) was able to induce embodiment in 40% of non-clinical participants. Interestingly, there was a significant decrease in embodiment when tactile stimulation was applied only to their own (unseen) arms whilst watching the mannequin’s arms from a first-person perspective [14]. This indicates that the influence of visual capture on embodiment did not result from social desirability or confabulation [14], the production of false memories without the purpose to deceive [15].

**Table 2:** Details of studies that used tactile-less embodiment methods in non-ED populations

Authors	Research question(s)	Participants	Embodiment process and measures	Main findings
Carey et al. [14]	How does simply observing a mannequin body from a first-person perspective affect subjective embodiment?	Experiment 1 *N* = 40; females; Age: *M* = 20.15 years Experiment 2 *N* = 40; females; Age: *M* = 18.98 Location: UK	Participants observed a mannequin body via a head-mounted display from a first-person perspective Embodiment measured via a subjective embodiment questionnaire	40% of participants experienced embodiment
Estudillo and Bindemann [21]	Can gaze-contingent mirror-feedback from unfamiliar faces alter self-recognition?	*N* = 13; females; Age: *M* = 22.0 years 0 Location: UK	Participants’ eye movements were mimicked by an onscreen stimulation face Enfacement measured via a subjective enfacement questionnaire and objective self-other discrimination morphing task	Participants reported a subjective experience of embodiment, but the stimulation did not affect their perceptual self-representations as measured by self-other discrimination morphing task
Ma et al. [23]	Does enfacing a virtual face also include the emotion of the face?	*N* = 20 (9 females); Age: *M* = 22.30 years Location: Netherlands	Participants saw a virtual face moving synchronously with their own within a VR setting Enfacement measured via a subjective enfacement questionnaire and Including Other in the Self scale	Enfacement illusion was successfully evoked in a VR environment (without tactile stimulation) Participants adopted the expressed emotion (i.e., enfacing a happy face improved mood)
Martinaud et al. [16]	Does visual capture without tactile stimulation by a rubber hand induce body ownership in hemiplegic patients?	*N* = 31 hemiplegic patients (16 females); Age: *M* = 68.35 years (calculated by the authors of this review as there was no information provided by the original authors) Location: UK	Hemiplegic patients observed a rubber hand for 15 s that was placed on a pillow (in a congruent position as their own hidden paralyzed hand) Embodiment measured via subjective rubber hand ownership questions and objective lesion analysis	A majority of the patients experienced strong ownership over the rubber hand (without tactile stimulation)
Serino et al. [22]	Does experiencing visuo-motor synchrony (without tactile stimulation) with a VR avatar's face make participants merge the face into their own face?	Experiment 1 *N* = 24; females; Age: *M* = 23.00 years Experiment 2 *N* = 16; females; Age: *M* = 24.00 years Location: Switzerland	Participants observed an avatar’s face moving in synchrony and asynchrony with their own face via 3D VR Enfacement measured via a subjective enfacement questionnaire and objective self-face recognition morphing task	Participants tended to recognise the avatar’s face as their own after synchronous exposure, which was assessed by both subjective and objective measures

*VR* Virtual Reality

**Table 3:** Details of studies that used tactile and tactile-less embodiment methods in ED populations

Authors	Research question(s)	Participants	Embodiment process and measures	Main findings
Eshkevari et al. [26]	Do people with EDs (AN, BN, or an eating disorder not otherwise specified [EDNOS]) report a stronger experience of the RHI than healthy controls (HCs)?	*N* = 139 females (AN = 36; Age: *M* = 23.00 years; BMI: *M* = 16.10) (EDNOS = 20; Age: *M* = 27.50 years; BMI: *M* = 19.70) (BN = 22; Age: *M* = 22.50 years; BMI: *M* = 20.90) (HC = 61; Age: *M* = 24.00 years; BMI: *M* = 21.5) Location: UK	Participants saw a rubber hand being stroked synchronously or asynchronously with their own unseen hand Embodiment measured via a subjective embodiment questionnaire and objective proprioceptive drift	Participants with EDs reported a significantly stronger experience of the RHI on both subjective and objective measures. Note: no comparison between ED groups reported by authors
Eshkevari et al. [1]	Do people who have recovered from an ED show differences in embodiment (RHI) when compared to current ED and HC participants?	*N* = 167 females (current ED and HC groups as per Eshkevari et al. [26] (Recovered ED = 28; Age: *M* = 25.5 years; BMI: *M* = 20.7) Location: UK	As per Eshkevari et al. [26]	Recovered and current ED participants experienced similar levels of subjective embodiment; both groups showed higher levels of subjective embodiment compared to HCs. For the objective measure, no difference was reported between any groups *Note*: there was a failure to replicate the finding between ED and HC groups for the objective measure, despite being the same sample as Eshkevari et al. [26] (likely due to insufficient power with the addition of the recovered ED sample, although rounded up scores differed between studies)
Keizer et al. [27]	Does the experience of ownership over a rubber hand change body size perception in AN participants?	*N* = 60 females (AN = 30; Age: *M* = 26.37 years; BMI: *M* = 17.5 and HC = 30; Age: *M* = 21.80 years; BMI: *M* = 21.19) Location: Netherlands	Participants saw a rubber hand being stroked synchronously or asynchronously with their own unseen hand Embodiment measured via a subjective embodiment questionnaire and objective proprioceptive drift. Hand size over-estimation was used as a measure for the effect of embodiment	AN participants showed stronger RHI than HCs when measured subjectively, but only in the synchronous condition. For proprioceptive drift, no difference between groups Reduced hand size over-estimation occurred in both synchronous and asynchronous conditions (compared to pre-embodiment) in AN participants only
Keizer et al. [28]	Does experiencing a FBI change the estimation of body parts that are more emotionally salient than the hand?	*N* = 59 females (AN = 30; Age: *M* = 22.03 years; BMI: *M* = 18.11 and HC = 29; Age: *M* = 21.07 years; BMI: *M* = 20.77) Location: Netherlands	Through VR goggles, participants observed a virtual avatar’s body (from a first-person perspective) being stroked in synchrony or asynchrony with their own unseen abdomen Embodiment measured via a subjective embodiment questionnaire. Objective body size estimation task (circumference and width of abdomen, hips, shoulders; body height) was used as a measure for the effect of embodiment	AN and HC groups did not differ in the strength of subjective embodiment. *Note*: no analyses were performed using the total subjective questionnaire; only with each subscale Experiencing a FBI decreased both AN participants’ and HCs’ over-estimation of all body parts, except for abdomen circumference (only AN participants showed a decrease in abdomen circumference over-estimates) Over-estimation changes from pre to follow-up (2 h and 45 min after the FBI) were larger in AN participants than HCs in shoulder width and circumference, and hip circumference estimations At follow-up after the FBI, AN participants shoulder size estimation was fully normalised, although a larger percentage of body size overestimations for the abdomen and hips were still present when compared to HCs
Malighetti et al. [40]	Can a VR induced FBI improve body satisfaction and body estimation accuracy, and decrease body image related concerns?	*N* = 7 females with AN (Age: *M* = 17 years; BMI: *M* = 15.95) Location: Italy	Participants experienced tactile-less visuo-motor synchronization with an avatar in the VR setting from first- and third-person perspectives. The BMI of the avatar corresponded to the participant’s real BMI at the beginning, but increased over successive sessions until it reached a healthy BMI Embodiment measured by a subjective embodiment questionnaire. Body size estimation task required participants to verbally specify how to change the avatar’s body to match it with their ideal and perceived real body sizes. Body satisfaction and image related concerns were assessed by questionnaires and scales pre and post VR intervention	Participants reported a trend of reducing body dissatisfaction and body image related concerns after VR intervention After the intervention, they preferred a body with a closer to normal BMI
Porras-Garcia et al. [29]	Can a VR induced FBI added to treatment as usual (TAU; including nutritional rehabilitation, group counseling, and cognitive-behavioural therapy) decrease the fear of gaining weight (FGW) and other body-related disturbances?	*N* = 35 females with AN (Age: *M* = 18.73 years; BMI: *M* = 17.42) Location: Spain	Participants were exposed to a virtual body with their real size and BMI, which slightly increased over following sessions until their healthy BMI target was reached. Both tactile-less visuo-motor and visuo-tactile stimulations were used Self-reported FBI and FGW levels were evaluated in the VR setting using a one item subjective analogue scale for the intensity of the FBI and FGW, respectively. Body image disturbances were assessed by several questionnaires pre and post intervention, and at follow-up	The experimental group who received both TAU and VR intervention had significantly lower levels of FGW and body image disturbances than the control group who only received TAU at follow-up (3 months) as well as after the intervention
Porras-Garcia et al. [30]	Can a VR induced FBI effectively measure key body-related cognitive and emotional responses in AN?	*N* = 73 females (AN = 30; Age: *M* = 17.73 years; BMI: *M* = 17.55 and HC = 43; Age: *M* = 21.12 years; BMI: *M* = 21.94) Location: Spain	Through a head-mounted display, participants observed a virtual avatar’s body (from a first-person perspective) being stroked in synchrony/asynchrony with their own unseen abdomen Embodiment measured via a one item subjective visual analogue scale for the intensity of the FBI	The level of FBI experience was significantly lower in AN participants than HCs. There was a significant negative relationship between FBI and body image disturbances (i.e., in people with AN, as disturbances increased, susceptibility to FBI decreased)
Provenzano et al. [2]	Does the experience of a VR induced embodiment illusion reduce body dissatisfaction in AN participants? Do AN and HC groups differ with respect to emotional reactions to experiencing embodiment?	*N* = 40 females (AN = 20; Age: *M* = 23.30 years; BMI: *M* = 15.87 and HC = 20; Age: *M* = 23.85 years; BMI: *M* = 18.98) Location: Italy	Through a head-mounted display, participants observed three virtual avatar’s bodies (from a first-person perspective) being stroked in synchrony or asynchrony with their own unseen abdomen. These avatars varied in relation to body size (lower than real BMI, real BMI, and larger than BMI). Each avatar was experienced one at a time Embodiment measured via subjective visual analogue scales. Affective reactions were measured using visual analogue scales in response to the experience of embodiment with each avatar. Dissatisfaction was measured using a perceived vs ideal body task (i.e., out of a series of avatars, participants chose those which they felt related to (i) their real size and (ii) their ideal size)	AN participants showed more negative response to the larger avatar used for embodiment compared to HCs. HCs preferred the larger rather than smaller avatar, whilst AN participants preferred the smaller rather than larger avatar AN and HC groups did not show any change in body dissatisfaction regardless of avatar body size
Serino et al. [39]	Does the illusion of owning a virtual body change body size estimation in AN participants?	*N* = 23 females suffering from AN; Age: *M* = 22.76 years; BMI: *M* = 15.50 Location: Italy	Participants watched a virtual body (abdomen) being stroked synchronously or asynchronously with their own abdomen through a head-mounted display. The illusion was delivered in two sessions, one before (T1) and one after (T2) experiencing treatment provided by a centre of excellence The effect of embodiment was measured via an objective body size (abdomen, shoulders, and hips) estimation task	There were significant decreases in the circumference estimation of the abdomen and hips only following the illusion delivered after the treatment (T2)
Serino et al. [38]	Can VR-based embodiment illusions reduce body image distortions in an AN participant?	One female with AN; Age = 30 s (no exact information for age provided); BMI = 13.69 Location: Italy	The participant observed a virtual abdomen of a healthy-weight woman being stroked in synchrony or asynchrony with her own abdomen through a head-mounted display. The illusion was delivered in three sessions over the course of her outpatient treatment (i.e., start, end, and 1 year follow up) Embodiment measured via subjective embodiment questionnaire. The effect of embodiment was assessed by an objective body size estimation task (abdomen, hips, and shoulders)	At the beginning of the treatment, she reported high scores for all three subscales of the embodiment questionnaire. No evidence for embodiment at end of treatment or 1 year follow-up, although she reported a high score for the asynchronous condition at follow up for the ownership subscale Body size estimations for width were reduced at the start of treatment for synchronous compared to asynchronous stroking (it should be noted her general tendency to overestimate body width even before embodiment happened). At the end of the treatment, experiencing embodiment did not reduce estimations involving width (i.e., she still showed a general tendency for overestimating width). At 1 year follow-up, experiencing embodiment generally did not influence width estimations (although her general estimations were already accurate), although she over-estimated the width of her hips more after asynchronous embodiment. Estimations for body circumferences showed similar patterns to those for width across time points In general, ‘more emotionally-laden sites’ such as the abdomen and hips were more susceptible to overestimation than shoulders throughout the procedure
Zopf et al. [31]	Do AN and HC groups differ in susceptibility to RHI?	*N* = 46 females (AN = 23; Age: *M* = 21.87 years; BMI: *M* = 15.82 and HC = 23; Age: *M* = 21.48 years; BMI: *M* = 21.16) Location: Australia	Participants saw a rubber hand being stroked synchronously or asynchronously with their own unseen hand Embodiment measured via a modified subjective RHI questionnaire and objective reaching task	AN participants experienced a stronger RHI than HCs when measured subjectively and objectively

*BMI* Body Mass Index, *ED* Eating Disorders, *AN* Anorexia Nervosa, *BN* Bulimia Nervosa, *EDNOS* Eating Disorder Not Otherwise Specified, *HC* Healthy Control, *RHI* Rubber Hand Illusion, *FBI* Full Body Illusion, *VR* Virtual Reality

Similar results have been observed in both non-clinical and clinical populations (e.g., hemiplegic patients; i.e., those who experience paralysis on one side of their bodies) when using tactile-reduced versions of the RHI paradigm (e.g., [16–18]). For example, as per Carey and colleagues [14], non-clinical participants experienced illusory ownership of a fake body by simply viewing it from a first-person perspective in a VR setting [19]. In another study involving hemiplegic patients, mere visual exposure to a motionless rubber hand for 15 s produced a strong sense of ownership over the hand corresponding to their paralyzed side [16]. In summary, these findings based on five studies suggest that tactile-reduced approaches may be an effective alternative means for producing illusory ownership over a fake body or body parts. Whilst such approaches have potential utility with the continued emergence of improved, yet cost affordable VR technologies, it is worth considering whether simpler (i.e., non-VR) approaches may also be viable options. One such option involves merely copying an actor outside of a VR setting.

### Using synchronic mimicry as a tactile-reduced approach to induce illusory embodiment

Copying of another person’s actions (via either mimicry or imitation) has been found to elicit ‘self-other overlap’ effects that can encourage prosocial phenomena such as increasing empathy and reducing prejudice (e.g., [20]). Given such findings, it is unsurprising that an increasing number of studies have employed copying approaches as a tactile-reduced method to elicit embodiment experiences. Specifically, researchers have attempted to evoke the enfacement illusion by combining visual cues with either synchronous facial or head movements ([21, 22]; see Table 1 for a description of common measures used in enfacement research). For instance, when healthy participants’ own eye-gaze movements were mimicked by the onscreen model at the same time, they reported an increased subjective feeling that the onscreen model’s face was their own when compared to an asynchronous mimicry condition [21]. However, unlike enfacement studies that have used the classic approach (with tactile input), synchronic facial mimicry did not effect participants’ enfacement when measured objectively (i.e., using a self-other discrimination morphing task; see Table 2 for details) [21]. The authors explained that this difference could be attributed to the short stimulation phase (i.e., two minutes) which was not enough to modify participants’ self-representations which had been accumulating for over 20 years, making them less susceptible to modifications involving other identities [21]. We note, however, that such an explanation appears to be contradictory to their claim involving the subjective measure (where enfacement was reported despite a short procedure). It is also worth noting that the reliability and hence validity of the self-other morphing tasks used in this study have yet to be established (e.g., it is unclear whether participants will choose a similar image as ‘self’ on different days, using different ‘other’ models who vary in attractiveness, etc.). More broadly, there has been little attempt in the embodiment literature to discuss the relationship between subjective and objective measures (see below for further discussion).

Facial mimicry has also been investigated via VR using a ‘mirror exposure’ paradigm, which demonstrated that the enfacement illusion was elicited—both subjectively and objectively—when participants observed avatars that moved synchronously with their own head/facial movements (e.g., rotation movements of the head) [22, 23]. Clearly, tactile-reduced approaches involving synchronic copying, such as enfacement, appear to offer a somewhat simplified means for studying embodiment illusions. However, before we elaborate on the advantages of such an approach, we will first consider how ED patients themselves may experience embodiment illusions and whether there may be any potential benefits of doing so.

### Embodiment illusions in eating disorder patients

ED patients typically show issues involving self-perception such as having body image concerns, poor appearance satisfaction (or high appearance dissatisfaction), and/or a tendency to see themselves as being bigger than they really are [24]. One possible explanation for this is the Allocentric Lock Theory [25], which suggests that ED patients have an insufficient *memory* regarding the appearance of their bodies that leads to a distorted experience of their *actual* bodies [25]. Specifically, patients are “locked” to an allocentric (i.e., a third-person) perspective based upon their (false) memory of themselves being fat, whilst the current actual body viewed from an egocentric (i.e., first-person) perspective is extremely thin. Stated differently, the inability for ED patients to update their allocentric memory using egocentric representations formed by bodily perceptions disturbs how their bodies are experienced and remembered [25].

Such characteristics indicate that ED and non-ED populations may show differences involving the multisensory integration processes underlying embodiment illusions and self-perception more broadly, as explained according to the previously outlined predictive coding theory [10, 11]. As indicated in Table 3, researchers have begun to employ the classic embodiment and tactile-reduced paradigms to study the extent to which embodiment illusions occur in ED patients (e.g., [1, 2, 9, 27–31]).

Out of the seven studies outlined in Table 3 which have included ED participants and healthy controls (HCs) published since 2012 (i.e., the year of the first paper we were able to identify), five have reported differences involving susceptibility to embodiment illusions [1, 9, 27, 30, 31]. Four of these five studies reported that ED participants showed *increased* susceptibility to embodiment [1, 9, 27, 31], whilst the fifth study reported that ED participants were *less* susceptible compared to HCs [30]. When considering how to account for this latter contradictory finding, some potential methodological issues are worthy of acknowledgement (e.g., only a single item visual analogue scale was used to assess the intensity of embodiment). Finally, two papers reported that no differences emerged between ED participants and HCs [2, 28]; although again, there are important methodological issues that may contribute to these null findings (e.g., no analysis was performed using the total score from subjective embodiment questionnaires, only with each subscale).

In summary, whilst still in its infancy, research investigating embodiment illusions in ED participants indicates that stronger embodiment illusions are experienced by ED participants than HCs. Such an increased susceptibility is likely the result of visual input being more dominant in ED patients during multisensory integration, due to impairments involving other sensory input. Specifically, people with EDs have been reported to show reduced proprioception (e.g., [32]) as well as reduced interoception (e.g., [33]), and impaired tactile sensitivity (e.g., [34]). This in turn may be useful information for better understanding various issues associated with self-perception in people with EDs as postulated by the Allocentric Lock Theory [25].

We can now consider whether experiencing embodiment illusions can lead to any improvements involving self-perception (e.g., increased body satisfaction, reduced body image concerns, and/or reduced body size overestimations, etc.). Research with non-clinical samples has provided some instances where embodiment led to improved body image-related concerns (e.g., [35–37]). For example, in one VR study that examined the pre versus post effects of experiencing an embodiment illusion, ‘healthy’ participants reported (i) decreases in hip size judgment and (ii) higher body satisfaction, after viewing a slimmer (but not a larger) avatar’s hips being stroked in synchrony with their own hips [37].

According to our search of the literature for such studies involving ED participants, there are at least seven studies that have been published since 2014 (the details of these studies are provided in Table 3; [2, 27–29, 38–40]). Encouragingly, six out of these seven studies have reported evidence for improvements involving image related concerns (usually as indicated via reduced size over-estimations). However, variation exists regarding the body parts targeted in each study [27–29, 38–40]. Amongst these studies, positive effects remained for at least two hours after experiencing the FBI [28]. The one study that reported no improvements for either ED participants or HCs nonetheless noted important differences involving the size (i.e., body mass index [BMI]) of the avatar used for establishing the embodiment illusions: whilst the HCs preferred large models rather than small models, the opposite was reported for ED participants [2].

To summarise, in addition to non-clinical research, a growing number of clinical studies have begun to establish the use of embodiment illusions as a means for improving image related concerns. Positive effects may be the result of participants combining their representation (or mental model) of their own appearance with a representation of the embodiment model (who likely has a healthy and/or desirable appearance). This updated combined self (with other) representation is less distorted as reflected by reduced indications of body image concern. We can further consider this explanation within the framework offered by Allocentric Lock Theory: embodying another person’s features from a first-person perspective can “unlock” them from their distorted allocentric memory about their appearance.

### The benefits of tactile-reduced embodiment research in people with EDs

The above research indicates that embodiment illusions may offer crucial information about (i) the nature of the multisensory mechanisms underlying distorted self-perception in people with EDs (and how these may relate to allocentric lock theory), and ultimately (ii) whether and how self-perception can be improved. Nonetheless, challenges remain. If embodiment techniques are useful or in fact the only means for researching and treating EDs via online environments (i.e., during pandemics or involving remote locations), one major pragmatic issue involves the need for an approach that does not involve a third party to induce embodiment illusions. In other words, research and treatments delivered online should avoid tactile input, as this would require a third person to help induce the illusion. Another major limitation of current VR interventions for EDs—in addition to accessibility and cost—is that they still require face-to-face contact with the researcher to operate the machine even if they do not use tactile stimulation need to be addressed. Methods involving a tactile-reduced approach—such as enfacement using the mimicry method—appear to be a valid alternative. Despite such benefits, however, to date researchers have rarely utilised a tactile-reduced approach among ED populations. Going forward we offer the following suggestions for using tactile-reduced embodiment approaches for studying ED populations in relation to (i) self-perception and (ii) possible interventions.

### Directions for future research: using embodiment illusions to understand self-perception in ED populations

Researchers have established some ‘best practice’ components that should be utilised in any future research that aims to investigate (or elicit) embodiment illusions. One such practice involves the need to ensure that embodiment illusions involve both synchronous and asynchronous timing procedures. The latter effectively serves as a control for the former and can help determine the extent to which the *timing* of multisensory processes is an important factor. Secondly, careful consideration should be given to the body parts being targeted when eliciting the illusion in ED participants. Across the studies reviewed here, embodiment illusions have been successfully induced irrespective of the body parts targeted, including the hands, faces, and abdomen (usually as an attempt to evoke FBI). Whilst this may indicate that the body part chosen to elicit the illusion may not matter, there is an absence of evidence involving an explicit attempt to compare body parts amongst the same sample. Such investigations are important given that ED participants have been found to consistently report body image distortions in highly salient body parts, such as the abdomen and hips [41]. However, it remains unclear whether these salient body parts show increased or reduced susceptibility to embodiment illusions.

Another potentially crucial factor involves the choice of model (or avatar) used to elicit the illusion. We note that very few studies have investigated or described the size of the models used (e.g., waist circumference, waist to hip ratio and/or the relative size [in percentages] of the model compared to the participant) [2, 28, 38, 39]. Amongst the few studies that have described model size, only one has manipulated the size of the model to see whether it influenced embodiment, with models showing characteristics either − 15%, 0%, and + 15% differences from the participant’s own BMI [2]: anorexia nervosa participants in this study rated the large embodiment model more negatively than the models considered to be smaller in size. Future research should consider the influence of different sized models on participants’ experience of embodiment and provide more details regarding the characteristics of the models used.

As for whether studies should involve real-life or VR approaches, again, both have shown promising results in inducing embodiment. Although current VR technology is considered slightly costly and inaccessible for many people, we encourage its further use and exploration as an alternative to situations in which accessing real-life models may be challenging, especially as the technology will inevitably become more affordable and accessible.

Perhaps most importantly, however, the measures used to determine whether embodiment occurs requires careful consideration. Subjective and objective measures offer contrasting sources of information and both should be used when possible. Amongst the papers reviewed here, at least nine studies have employed both types of measures [1, 9, 16, 21, 22, 27, 28, 31, 38]. It would be ideal for future researchers to better ascertain exactly how these measures relate to each other (e.g., how to interpret the results if there is subjective embodiment but not objective embodiment, or vice-versa). Moreover, there is a need to ensure greater consistency regarding the measures chosen and how they are reported. This is particularly so for subjective questionnaires which past researchers have tended to score and report differently. For instance, whilst some have reported total scores (e.g., [16, 23]), others have only reported single items (e.g., [30]), or sub-scales (e.g., [2, 27, 28]). This makes direct comparisons across studies difficult in terms of ensuring the same (or similar) constructs are being reported. Relatedly, insufficient detail in reporting statistical results (e.g., no effect sizes and/or means, etc.) impairs future attempts to fully compare and understand various findings within the literature.

### Directions for future research: using embodiment illusions to improve self-perception in ED populations

All of the factors described in the prior section also apply in terms of designing experiments to investigate the effects of experiencing embodiment illusions. However, a few issues should be reconsidered. For instance, whilst it is ideal to include synchronous and asynchronous timing conditions to elicit embodiment illusions, at least one study [27] has reported positive (and equal) effects for body parts size overestimation irrespective of timing. Nonetheless, until further replication has occurred, we believe our original advice above should be adhered to.

The body parts used may be a more important factor for reducing image-related concerns than eliciting an embodiment illusion per se. Amongst those studies that included measures for various parts of the body to be assessed, some showed less distortion and higher susceptibility to improvement (i.e., shoulders) whilst others were more distorted and less susceptible to improvement (i.e., abdomen and hips) [34]. This may or may not be related to the issue of saliency described above, and as such should be subject to further research before any concrete suggestions are proposed. One particularly salient area of the body—the face—has yet to be subjected to any investigation regarding whether it can result in improved image-related concerns. Enfacement, which can be elicited via mimicry (without VR), should therefore be of particular interest to future researchers.

The qualities of the model used also require further consideration when it comes to ensuring positive effects relating to image concerns can be induced. Again, we note the lack of reporting involving the detailed characteristics of the models’ body parts used, although healthy sized models have been able to induce positive effects [38]. The one study [2] that compared different sized models found—curiously—that model size did not influence changes in image related concerns. Again, it would be premature to offer any definitive guidance on which sized models may have the most positive impact, hence, warranting further research on this issue. One potential ethical issue, however, involves whether ED patients who may show extremely low (and medically dangerous) BMIs should be exposed to embodiment models who have similar (or lower) BMIs. Whilst an embodiment process involving such a model may result in a person having fewer concerns about their current size, it may also mean that such people may lose motivation to increase their otherwise (dangerously) low BMI. Such instances would be analogous to exposing people to images of ‘thin-ideal’ models, the negative effects of which have been well documented (e.g., [42, 43]). From our reading of the current literature, it appears that no embodiment studies involving ED participants have used such models; rather, we have inferred from available information that models tend to be within the lower or upper limits of a healthy BMI range. It remains unclear as to whether this is due to the concern we have raised here.

Finally, whilst there are several well-respected body image related measures, researchers must attempt to use a similar battery of such measures (ideally those with the best psychometric properties) to allow for better comparisons across studies.

### Clinical implications

Evidence-based treatments for EDs have significantly improved in quality and quantity over recent years, yet there is still room for improvement [44]. For instance, typically only 30–50% of ED patients report cessation of symptoms following cognitive behavioural therapy (CBT), with the remaining exhibiting either partial remission, no or minimal improvement, or premature withdrawal from treatment [45]. Whilst CBT holds utility in ED treatment, such limitations necessitate the development of alternative treatment approaches for ED patients, as well as prevention and early intervention strategies targeting those at high risk for developing an ED or having other serious body image concerns.

The use of embodiment procedures provides one effective means for doing so. Indeed, not only do these procedures provide new tools for better understanding the basis and nature of the various self-perception issues reported in people with EDs and/or other body image concerns, these illusions already show a capacity for improving one’s responses to their body image and other related ED symptoms. Moreover, novel online tactile-reduced embodiment procedures as discussed in this review (e.g., mimicry-based enfacement) offer enhanced practical application and clinical utility compared to traditional embodiment methods. These tactile-reduced methods would enable targeted populations to access interventions more widely outside of a face-to-face environment. As discussed above this is particularly important within the context of COVID-19, which has necessitated restricted face-to-face contact, and thus has increased the vulnerability of individuals with ED.

Although the body image-related long-term benefits of embodiment remain unclear and require investigation to see whether they may extend beyond 3 months, effective tactile-reduced embodiment procedures may ultimately hold clinical utility as a complementary process for currently established interventions for ED treatment and/or prevention such as CBT. For example, to assess the effectiveness of such a novel approach, future studies should employ randomised controlled trials using ED and/or high-risk ED populations to compare a pure CBT approach (the control group) with a combination of CBT and embodiment (the experimental group). Such research is expected to enhance clinical practice that targets body image concerns in ED and related populations. To the best of our knowledge, only one study has reported the effectiveness of the combined intervention compared to treatment as usual only (i.e., nutritional rehabilitation, CBT and group counselling) at a 3-month follow-up [29].

## Conclusion

The current COVID-19 pandemic has raised an unanticipated and important question on how ED research and clinical practice targeting body image concerns should be conducted when face-to-face contact is restricted. Tactile-reduced embodiment illusions not only enable self-perception research to continue during such situations, but can also be utilised as a potential complementary intervention in ED and related populations with body image disturbances. This is supported by existing research that has consistently reported the ability of tactile-reduced techniques to generate embodiment illusions in non-ED populations, and the decrease in body overestimation in ED populations after experiencing either classic or tactile-reduced embodiment illusions. Though in its early stage, tactile-reduced embodiment illusions are expected to further enhance ED research and clinical practice with future studies investigating several aforementioned factors (e.g., embodiment model size, subjective and objective measures etc.).

## Data Availability

The data presented in the current review are all included in the tables.

## Abbreviations

AN: Anorexia nervosa
BMI: Body mass index
BN: Bulimia nervosa
CBT: Cognitive behavioural therapy
ED: Eating disorder
FBI: Full body illusion
HC: Healthy control
RHI: Rubber hand illusion
VR: Virtual reality
